# Nitric Oxide in Plants: The Roles of Ascorbate and Hemoglobin

**DOI:** 10.1371/journal.pone.0082611

**Published:** 2013-12-20

**Authors:** Xiaoguang Wang, Mark S. Hargrove

**Affiliations:** Department of Biochemistry, Biophysics, and Molecular Biology, Iowa State University, Ames, Iowa, United States of America; Albany Medical College, United States of America

## Abstract

Ascorbic acid and hemoglobins have been linked to nitric oxide metabolism in plants. It has been hypothesized that ascorbic acid directly reduces plant hemoglobin in support of NO scavenging, producing nitrate and monodehydroascorbate. In this scenario, monodehydroascorbate reductase uses NADH to reduce monodehydroascorbate back to ascorbate to sustain the cycle. To test this hypothesis, rates of rice nonsymbiotic hemoglobin reduction by ascorbate were measured directly, in the presence and absence of purified rice monodehydroascorbate reductase and NADH. Solution NO scavenging was also measured methodically in the presence and absence of rice nonsymbiotic hemoglobin and monodehydroascorbate reductase, under hypoxic and normoxic conditions, in an effort to gauge the likelihood of these proteins affecting NO metabolism in plant tissues. Our results indicate that ascorbic acid slowly reduces rice nonsymbiotic hemoglobin at a rate identical to myoglobin reduction. The product of the reaction is monodehydroascorbate, which can be efficiently reduced back to ascorbate in the presence of monodehydroascorbate reductase and NADH. However, our NO scavenging results suggest that the direct reduction of plant hemoglobin by ascorbic acid is unlikely to serve as a significant factor in NO metabolism, even in the presence of monodehydroascorbate reductase. Finally, the possibility that the direct reaction of nitrite/nitrous acid and ascorbic acid produces NO was measured at various pH values mimicking hypoxic plant cells. Our results suggest that this reaction is a likely source of NO as the plant cell pH drops below 7, and as nitrite concentrations rise to mM levels during hypoxia.

## Introduction

**Figure 1:**
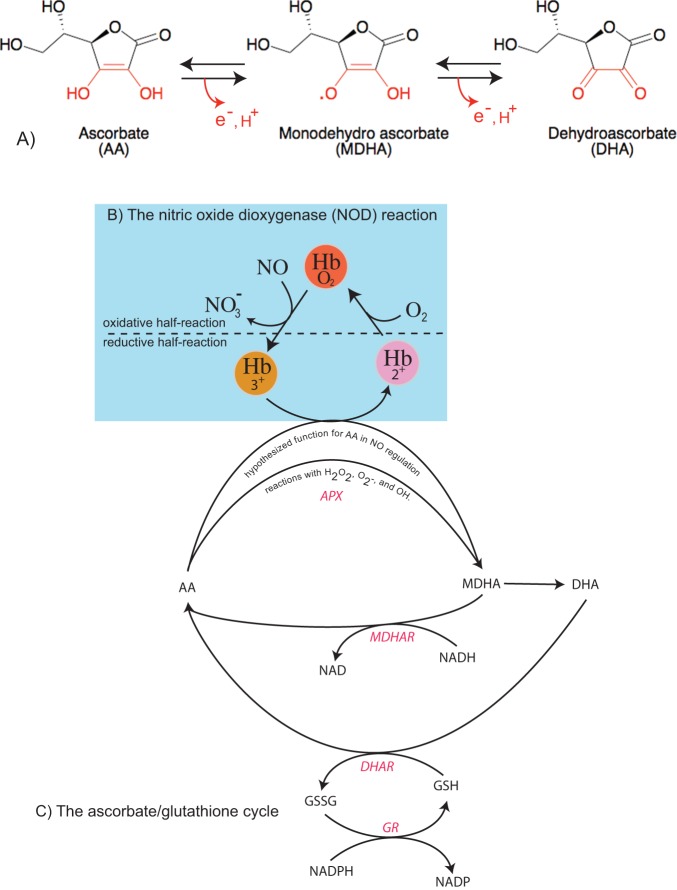
Pertinent chemical reactions of AA, hemoglobin, and NO. A) Ascorbic acid (AA) can lose an electron to become monodehydroascorbate (MDHA) radical, which can lose an electron to become dehydroascorbate (DHA). B) The nitric oxide dioxygenase (NOD) reaction; oxy hemoglobins (Hb) will react with NO to make nitrate and ferric Hb. If the ferric Hb is reduced (in this case by AA), it will bind oxygen (if present) to start the reaction again. C) The AA/glutathione cycle in plants is associated with scavenging of peroxide, superoxide, and hydroxyl radical, and is hypothesized to reduce nsHbs in support of NOD.

Ascorbic acid (AA) is an abundant and multifaceted biomolecule found in most living organisms [1], [2], [3]. The L-stereoisomer known as “vitamin C” is a cofactor for enzymatic reactions in animals that are important in collagen synthesis and wound repair. Additionally, humans normally maintain AA concentrations in the blood near 70 µM for the purpose of general antioxidant activity. These properties stem from the facile redox chemistry linking AA, monodehydroascorbic acid (MDHA), and dehydroascorbic acid (DHA) (Figure 1A).

The antioxidant role of AA in plants is evident from the relatively high concentrations found in many tissues [4]. On average, the AA concentration in *Arabidopsis* cells is 5.6 mM [5], and it is found in many sub-cellular organelles including mitochondria, peroxisomes, vacuoles, cytosol, the cell wall, and chloroplasts, where concentrations can reach 50 mM [4], [6], [7], [8], [9], [10]. Such high concentrations in metabolically active areas indicate an important role for AA in the defense against damage by reactive oxygen species (ROS). AA is also important for mitigating damage by ROS produced in hypoxic and anoxic roots, which plants experience in a variety of soil conditions [11], [12].

### The AA-glutathione cycle in plants

Plants contain a relatively robust mechanism for exploiting the antioxidant properties of AA, known as the AA-glutathione cycle [13], [14] (Figure 1C). In this cycle, AA reduces hydrogen peroxide (via AA peroxidase [15], APX), superoxide, and hydroxyl radical, each a product of excess reducing power in the cell. In these reactions, AA is initially oxidized to MDHA [16], [17], but DHA eventually results from spontaneous disproportionation of MDHA to AA and DHA [18]. The purpose of the cycle is that MDHA and DHA are much less reactive than superoxide, hydroxyl radical, and hydrogen peroxide, and the cell is granted time to dissipate chemical energy through less damaging mechanisms. The AA-glutathione cycle is completed by reduction of MDHA and DHA back to AA. Reduction of DHA is driven by reduced glutathione via the enzyme DHA-reductase (DHAR: EC 1.8.5.1), and reduction of MDHA is coupled to NAD(P)H oxidation by the enzyme MDHA-reductase (MDHAR: EC 1.6.5.4).

### Nitric Oxide

Another free radical ROS produced in plants and animals is nitric oxide (NO), which has diverse and important signaling functions and is often produced during hypoxia [19], [20]. NO and AA have even longer histories in the food sciences, where nitrate, nitrite, and AA are used in curing batter to give cured meat its characteristic deep red color [21], [22]. The cause of the color is NO, which binds hemoglobin and myoglobin and mimics the color of fresh meat. The source of NO in this reaction is the direct reduction of nitrous acid (protonated nitrite) by AA.

The origins of NO in hypoxic plant cells are not completely clear [23], but it is evident that significant NO concentrations are produced in these environments [20], [24]. The cytoplasmic pH of hypoxic plant cells can drop to pH 6.5 or lower [25], [26], while at the same time nitrite can rise to mM concentrations [23], [27] in the presence of similar levels of AA. These conditions are well-suited for NO formation from nitrite as a result of direct reduction by AA. However, this possibility has not yet been addressed directly in the context of plant physiology.

### Hemoglobins and NO scavenging

Bacteria and some yeast have a specific mechanism for scavenging NO called the NO dioxygenase (NOD) reaction (Figure 1B), in which NO is oxidized to nitrate via an oxygen-dependent reaction catalyzed by the enzyme flavohemoglobin [28], [29]. Flavohemoglobin contains a heme domain that binds oxygen, and a reductase domain that can reduce the heme using electrons from NAD(P)H. Oxy-flavohemoglobin reacts rapidly with NO, forming nitrate and ferric hemoglobin. The reductase domain then reduces the heme, which binds oxygen to complete the cycle (Figure 1B).

No such specific mechanism for NO scavenging has yet been affirmed in plants or animals, but it has been hypothesized that nonsymbiotic plant hemoglobins (nsHbs) might participate in this function. This hypothesis is based largely on the ability of oxy-nsHb to carryout the oxidative half-reaction with NO (Figure 1B; [30]), and indirectly on increased nsHb expression in several species in response to nitrate, nitrite, and nitric oxide [31], [32], NO scavenging in several plant species over-expressing nsHb [33], [34], [35], decreases in the levels of NO-sensitive enzymes in plants with down-regulated nsHb [36], and increases in NO emission from plants with down-regulated nsHb [24]. However, none of this work characterizes the reductive half of the NOD reaction, consisting of the one electron reduction of ferric nsHb (Figure 1B).

nsHb reduction is considered to be the rate limiting step for NO scavenging using the NOD mechanism [37]. Several mechanisms of nsHb reduction have been proposed including specific reductases, free flavins [38], or ascorbic acid [39]. The latter mechanism proposes that AA, with help from MDHAR, might serve as the reducing agent for ferric nsHb in the reductive NOD half-reaction (Figure 1B). In this scenario, AA directly reduces ferric nsHb, forming MDHA. MDHAR then uses NAD(P)H to reduce MHDA back to AA, thus coupling the reducing power of NAD(P)H to nsHb reduction in support of NOD function. There are some questions remaining whose answers would provide an important test of the hypothesis. 1) How fast is nsHb reduced by AA? The crux of this hypothesis rests on the ability of AA to reduce nsHb in an efficient manner, yet this reaction has not been measured directly. 2) How does MDHAR affect nsHb reduction by AA? 3) Does the AA/MDHAR system improve nsHb-mediated NO scavenging compared to the AA cycle alone? Finally, a very basic question regarding AA and NO must be answered in order to clarify the origins of NO: 4) Can AA directly reduce nitrite to form measurable NO levels under conditions mimicking hypoxic plant cells?

The experiments presented here address these questions by measuring the kinetics and products of AA reduction of rice nsHb with and without a purified MDHAR, and by directly measuring NO scavenging by each component of this system in controlled *in vitro* systems. Also measured is the direct production of NO by AA and nitrite as a function of pH, in an effort to gauge the likelihood of this reaction serving as an origin of NO in hypoxic plant cells.

## Materials and Methods

### Protein Production

*Oryza sativa* (rice) class 1 nonsymbiotic hemoglobin (nsHb) expression and purification was performed as described previously [40]. The cDNA sequence for rice cytosolic monodehydroascorbate reductase (MDHAR) was obtained from GenBank (D85764.1), and used to design PCR primers (Table 1). Single stranded *Oryza sativa* cDNA was produced by RT-PCR (Superscript III reverse transcriptase, Invitrogen, #18080-093) from total rice mRNA (a gift from Dr. Reuben Peters, Department of BBMB, Iowa State University), and oligo dT as a primer. Double stranded MDHAR cDNA was then amplified by PCR using the single stranded cDNA as a template, RCMD F and RCMD R as primers (Table 1). PCR primers RCMD NheI F and RCMD HindIII R were used to add NheI (5′) and HindIII (3′) restriction sites flanking MDHAR cDNA by another round of PCR. MDHAR cDNA was then cloned into pET-28a (Novagen, #69864–3) between the NheI and HindIII sites.

**Table 1:** Primers.

Primer Name	Sequence
RCMD F	5′-ATGGCGTCGGAGAAGCACTTC-3′
RCMD R	5′-TCATATTTTGCTGGCGAACTGGAGG-3′
RCMD NheI F	5′ACAACAACACGCTAGCATGGCGTCGGAGAAGCACTTC-3′
RCMD HindIII R	5′ACAACAACACAAGCTTTCATATTTTGCTGGCGAACTGGAGG-3′
EcoRV Fdx F	5′-ACAACAACACGATATCATGGCTGATTGGGTAACAGGCAAAG-3′
BamHI Fdx R	5′ACAACAACACGGATCCGGAGCTCGAATTCTTACCAGTAATGCTCC-3′

The His-tagged MDHAR gene was expressed [16], [41] in *E. coli* BL21(DE3)-RIPL cells (Stratagene, #230280). The cells were grown in LB medium at 37°C until the OD_600nm_ reached 0.6, then expression was induced with 0.5 mM IPTG and the temperature lowered to 25°C for 3 hours. The cells were lysed, and the enzyme was purified by Ni-NTA chromatography (Qiagen, #30210). Purified enzyme (determined as a single band by SDS-PAGE) was dialyzed against 50 mM phosphate buffer (pH  = 8.0) and concentrated with an Amicon Ultra-15 Centrifugal Filter Unit (Millipore, # UFC901008). MDHAR concentration was estimated based on its flavin absorbance using the extinction coefficient of 11.3 mM^−1^ cm^−1^ at 450 nm [42].

*E. coli* Ferredoxin-NADP reductase (Fdr) (GenBank: AAA23805.1) was expressed from a pET-28a vector (Novagen, #69864–3). Fdr was purified and quantified using the procedure described above for MDHAR.

### MDHAR assays

MDHAR enzyme activity was measured by monitoring the decrease in NADH absorbance at 340 nm [16]. A Varian Cary 50 Bio UV/Visible Spectrophotometer with Cary WinUV Kinetics software (Agilent Technologies) was used for data collection. The total reaction volume was 1 ml, containing 50 mM phosphate buffer, pH 8.0. (This pH was chosen for our MDHAR experiments to be consistent with previous work leading to this hypothesis [39].) 0.2 mM NADH was first added into a cuvette with 1 ml phosphate buffer, followed by AA from a freshly prepared stock to generate 3 mM AA in the cuvette, and 0.125 U/ml ascorbate oxidase (AO) (Sigma-Aldrich, # A0157). 1.22 µM or 0.024 µM DHAR were added to start the reaction. All data were plotted and analyzed using IGOR PRO software.

### Rice nsHb reduction assays

Rice nsHb reduction assays were carried out by monitoring the increase in absorbance at 575 nm in air, or 415 nm in CO saturated buffer, using methods described previously [37]. CO-saturated (1 mM) buffer was prepared by bubbling CO for 20 minutes into 50 mM phosphate buffer pH 8.0 contained in a 20 ml glass syringe. All assays were conducted in a cuvette with 1ml of 50 mM phosphate buffer, pH 8.0. For the reactions with high AA concentration (3, 6, 12 mM), 5 µM nsHb was added. For reduction experiments with excess nsHb, AA concentration was 2 µM and nsHb concentration was 10 µM. When NADH and MDHAR were involved, final concentrations were 0.2 mM and 0.73 µM respectively.

### EPR measurements of monodehydroascorbate

EPR spectra were collected with a Bruker ESP 300 spectrometer in Dr. Yeon-Kyun Shin's laboratory (Department of Biochemistry, Iowa State University). All spectra were collected at room temperature with the same instrument settings: modulation frequency, 100 kHz; modulation amplitude, 0.71 G [43]; time constant, 1310.72 ms; sweep time, 41.94 s; center field, 3298 G; sweep width, 7 G. All EPR samples were prepared by premixing reagents with 50 mM phosphate buffer (pH 8.0) to a total volume of 10 µl. Concentration of reagents (when involved) were: AA, 3 mM; NADH, 0.2 mM; MDHAR, 0.73 µM; AO, 0.0125 U/µl. Fresh AA was prepared right before experiment, while equilibrated AA was prepared and set at room temperature for 72 hours before experiment. All samples were 6 µl in round capillaries (Vitrotubes, # CV6084).

### NO scavenging and production experiments

Solution NO concentrations were measured with an ISO-NO MARK II Nitric Oxide Meter and Duo-18 recording system with Duo-18 software (WPI), which has been described previously [37]. A saturated NO solution (2 mM NO) was prepared by bubbling 50 mM phosphate buffer (pH 8.0) with ultra high purity N_2_ gas for 20 minutes, followed by bubbling with ultra high purity NO for 10 minutes. The NO was sparged through 5M NaOH on its way into the phosphate buffer. To prepare the sample, 1.95 ml of 50 mM phosphate buffer (pH 8.0) was first added into an electrode chamber, which was then sparged with ultra high purity N_2_ for 5 minutes for conditions simulating hypoxia. A FOXY-R oxygen electrode and OOISensors software (Ocean Optics, Inc.) were used to measure the oxygen concentrations during the hypoxic experiments. The oxygen electrode was calibrated with two points; 1M sodium dithionite solution (in 50 mM phosphate buffer, pH 8.0) was used as 0 µM oxygen, and 50 mM phosphate buffer (pH 8.0) bubbled with air for 10 minutes was used as 262 µM oxygen. Based on this calibration, our “hypoxic” conditions are 0.1 µM in oxygen.

To begin the NO scavenging experiments, reagents were added into the chamber for a premix to establish a baseline, and then a final concentration of 50 µM NO (50 µl of the saturated NO solution) was added to start the reaction. Reagents were (when used in the experiment): 3 mM AA, 3 mM DHA, 0.2 mM NADH, 5 µM rice nsHb, 0.73 µM MDHAR, 0.73 µM FNR, and 0.125 U/reaction ascorbate oxidase. Time course data were graphed with IGOR PRO software. Experiments measuring NO production from nitrite at various pH values were conducted by addition of 3 mM AA into 2 ml of 10 mM or 200 mM sodium nitrite (in the hypoxic NO electrode chamber) made in 50 mM phosphate buffer of varying pH. Sodium nitrite solutions in all experiments were first verified for pH and bubbled with ultra high purity N_2_ for 5 minutes.

### Statistical analysis and figure production

**Figure 2:**
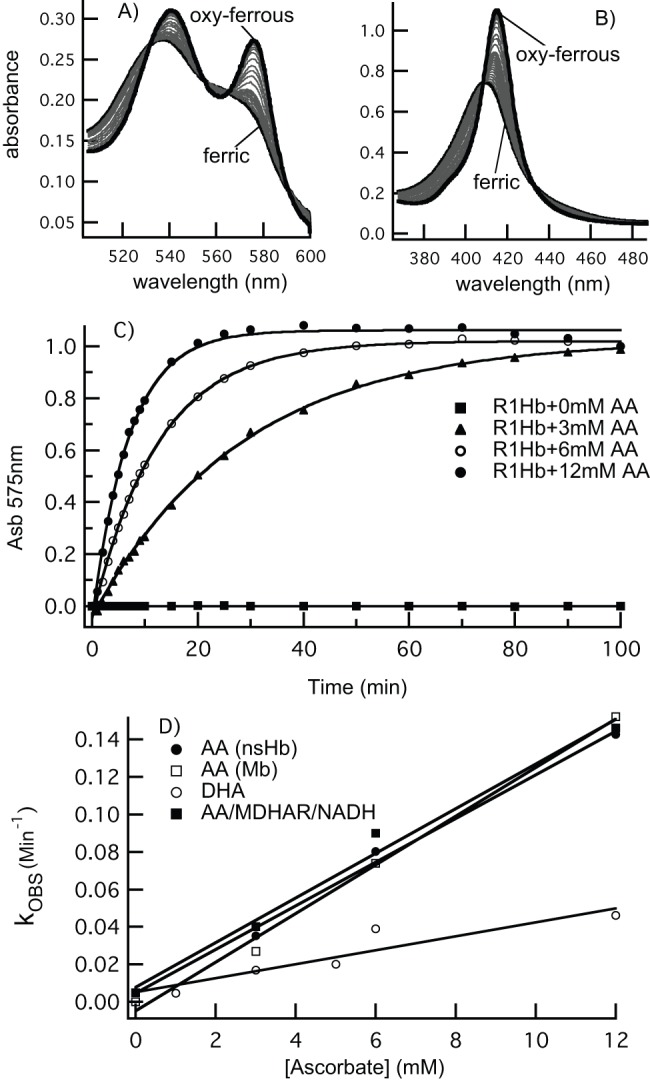
nsHb reduction by AA, DHA, and MDHAR. A) ferric rice nsHb (5 µM) was mixed with 3 mM AA in air-saturated buffer. The absorbance changes in the visible and B) Soret regions are associated with nsHb reduction and oxygen binding. C) Time courses for nsHb reduction by various concentrations of AA. D) The rate constants for AA reduction of nsHb and Mb (5 µM) are plotted as a function of AA concentration, which have slopes equal to the bimolecular rate constants for reduction of nsHb.

The spectra presented in Figures 2A and 2B are one example out of four independent replicates. Likewise, the time courses for NO scavenging are examples of at least four independent replicates, analysis of which led to the values in Table 1. The values in Table 1 are averages of at least four independent replicates. The absolute variance across the individual observations for each value in Table 1 was within 10% of the average. All data were plotted and analyzed using IGOR PRO software, and figures were produced in Adobe Illustrator.

## Results

### Rice nsHb is reduced directly by AA and DHA

A reaction key to the NOD function for AA and nsHb is reduction of nsHb by AA. Previous investigations of AA, MDHAR, and nsHb interactions inferred reduction of the Hb from measurements of NO scavenging by root extracts and partially purified nsHb fractions, and NADH consumption by a purified by MDHAR [39]. Neither set of experiments, however, directly measured nsHb reduction by AA.

Results of the reaction of AA with rice nsHb are presented in Figure 2. The starting (ferric) and final (oxy-ferrous) spectra are labeled, and demonstrate an isosbestic transition between the two states. Figure 2C are time courses for rice nsHb reduction at different AA concentrations, showing an increase in rate at higher AA concentrations expected for a bimolecular reaction. Rate constants for these time courses, and for those associated with rice nsHb reduction by DHA are shown in Figure 2D as function of AA concentration. Also included in Figure 2D are rate constants for the reduction of horse heart Mb, which were measured to gauge the specificity of the reaction for rice nsHb. In all cases, the rate constants are linearly dependent on AA and DHA concentrations, and fits yield bimolecular rate constants of 0.01 mM^−1^min^−1^ for nsHb and Mb, and 0.003 mM^−1^min^−1^ for reduction of nsHb by DHA.

**Figure 3:**
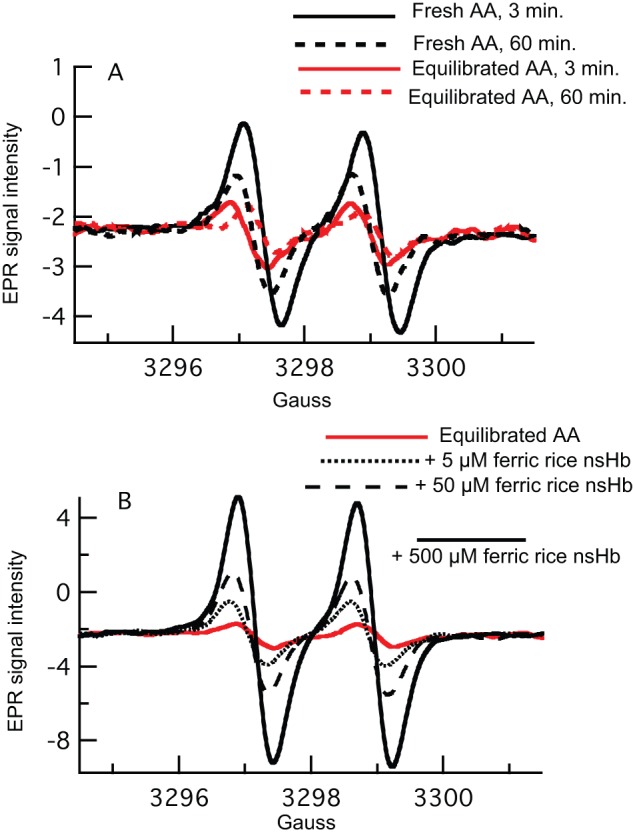
EPR measurements of MDHA radical in solutions of AA (A), and produced from AA reduction of nsHb (B). A) Freshly dissolved 3 mM AA solutions contain MDHA, which decays to a lower equilibrium level over several hours. B) Addition of ferric nsHb to AA increases the EPR signal associated with MDHA.

Reduction of a ferric Hb to the ferrous oxidation state requires one electron. If the reduction is coupled to AA oxidation, the initial product is expected to be the MDHA radical. As most of the experiments in this article involve redox reactions with AA, it is prudent to characterize the chemical state of AA in our solutions, and document product formation. For MDHA, this is accomplished by measuring the room temperature EPR signal associated with the MDHA radical [43]. When 3 mM AA is freshly dissolved, the solution initially contains MDHA on the order of 10 nM [43] (Figure 3A). The MDHA signal decays slowly (over hours) to an “equilibrated” value of ∼1 nM, which is stable indefinitely. Addition of ferric rice nsHb to equilibrated AA results in an increase in the MDHA EPR signal (Figure 3B). Thus, as expected for a one-electron Hb reduction by AA, it results in the production of MDHA.

### The role of MDHAR in nsHb reduction by AA

**Figure 4:**
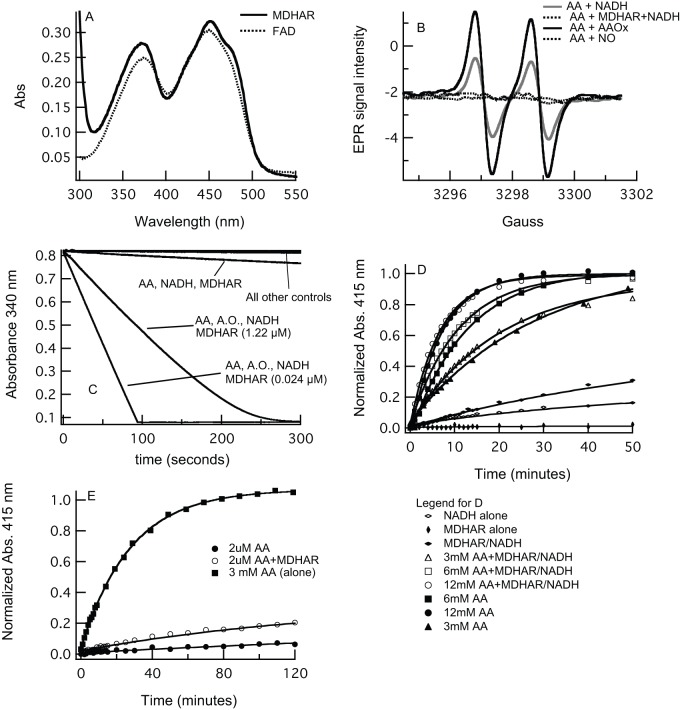
The effect of MDHAR on AA reduction of nsHb. A) Recombinant rice MDHAR was purified and quantified based on its flavin absorbance spectrum. B) The EPR signal associated with MDHA was used to demonstrate the activity of AAOx (which increases the MDHA signal in the presence of AA), and MDHAR (which decreases MDHA in the presence of NADH). These data also show that NO reacts directly with MDHA. C) MDHAR oxidizes NADH in the presence of MDHA (produced from AA and AAOx, as in (B)). D) Time courses for nsHb reduction by AA in the absence and present of MDHAR and NADH are nearly identical. E) When nsHb concentration is greater than AA, reduction is very slow, and slightly faster in the presence of MDHAR and NADH.

To test the effects of MDHAR on AA-mediated nsHb reduction, an MDHAR from rice was expressed using recombinant methods and purified to a single band as measured by SDS-PAGE. Rice MDHAR had the expected molecular mass of 46.6 kD, and purified from bacteria with a visible absorption spectrum characteristic of a flavoprotein (Figure 4A). Two experiments confirmed that our purified rice MDHAR contained the expected MDHA reductase activity. In the first, addition of MDHAR to a 3 mM solution of fresh AA (in the presence of NADH) completely removed the EPR signal associated with MDHA (Figure 4B). EPR was also used to confirm that AAOx produces MDHA from AA in our reaction conditions, so that we could be confident of substrate production for the reactions in Figure 4C. In the second experiment, MDHAR oxidized NADH only in the presence of MDHA (produced from AA and AAOx), and at rates proportional to MDHAR concentration (Figure 4C). Thus, our purified rice MDHAR has the expected ability to couple MDHA reduction to NADH oxidation.

Two experiments were also used to measure the effects of MDHAR on nsHb reduction by AA. In the first (Figure 4D), time courses for nsHb (5 µM) reduction were measured in the presence of excess AA (3 to 12 mM), with and without MHDAR and NADH. MDHAR alone (without AA or NADH) did not reduce nsHb at all, whereas NADH alone (or with MDHAR) reduced nsHb very slowly. In the presence of AA, reduction was accelerated to the levels observed in Figure 2C, but were not affected at all by the inclusion of MDHAR and NADH. These reduction rate constants are plotted along with those by AA alone in Figure 2D, and their linear fit yields a bimolecular rate constant of 0.01 mM^−1^min^−1^, identical to that by AA alone.

The second experiment tested nsHb reduction when AA concentration (2 µM) is lower than nsHb (10 µM). Under these conditions one might expect that the benefit of MDHAR/NADH would become evident, as the AA must be cycled back from MDHA to complete the reaction. Figure 4E demonstrates a measurable effect of MDHAR, but the time courses for reduction are very slow (as expected from this AA concentration).

### nsHb-driven NO scavenging: the effects of AA and MDHAR

The effects of AA, MDHAR, and nsHb on NO scavenging in solution were tested using an NO-specific electrode. In each experiment, the test conditions were equilibrated to establish a stable baseline, and NO scavenging was initiated by the addition of 50 µM NO to the reaction cell. NO is only intermediately stable, as it reacts with itself and other molecules in solution, especially oxygen, over a period of several minutes [44], [45]. Time courses for NO removal are complex and do not obey simple linear or exponential decay patterns. Thus, we have chosen the time it takes for 80% of the NO signal to decay (t_NO_, Table 2) as our objective measure for the purpose of comparing different experimental conditions.

**Table:** Table 2. NO scavenging reactions.

Reactions with Asc, DHA, MDHAR, and Asc Oxidase.							
	Buffer	Asc	DHA	NADH	NADH	MDHAR	AscOx
					Asc	NADH	Asc
						Asc	
Air	2.5	2.4	1.6	2.0	2.7	3.2	1.9
Hypoxia	16	>30	4	19	>30	>30	>30

Reactions involving nsHb1.							
	nsHb1	nsHb1	nsHb1	nsHb1	nsHb1	nsHb1	
		Asc	NADH	Asc	Asc	FdxR	
				NADH	NADH	NADH	
					MDHAR		
Air	2.9	2.4	2.7	2.4	3.0	1.1	
Hypoxia	27	36	16	26	29	10	

The time (in minutes) at which 80% of signal associated with 50 µM NO has been depleted (t_NO_) is listed at various reaction conditions.

**Figure 5:**
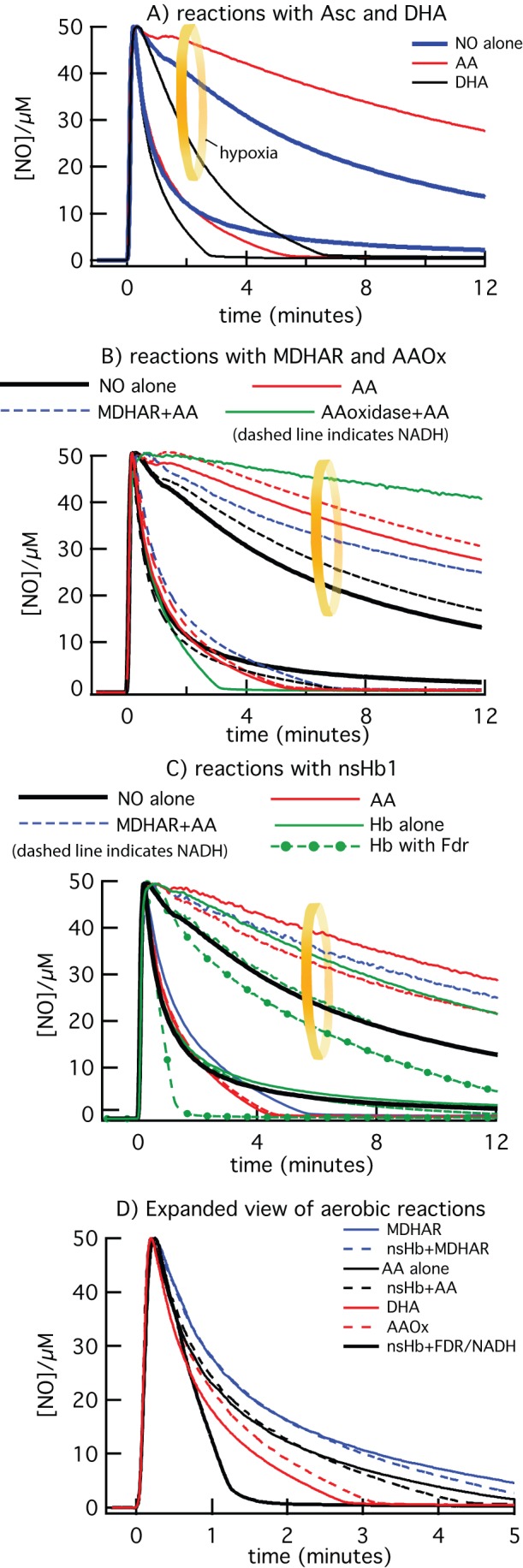
NO scavenging by AA, nsHb, and MDHAR. A) NO scavenging by buffer, AA, and DHA are compared under aerobic and hypoxic (0.1 µM [O_2_], inside the yellow circle) conditions. B) NO scavenging with MDHAR and AAOx under aerobic and hypoxic (inside the yellow circle) conditions. The curves for NO alone are black, and the presence of AA (red), MDAHR and AA (blue), and AAOx (green) are indicated by color. A dashed line indicates the presence of NADH (0.2 mM) in that reaction condition. C) NO scavenging by nsHb, with and without MDHAR are compared to that facilitated with reduction of nsHb by ferredoxin reductase (Fdr, green dashed line with filled circles, indicating Fdr and NADH are present along with nsHb). D) An expanded view of aerobic NO scavenging demonstrates the subtle effects of AA and DHA.

To establish a baseline for NO decay time courses, Figure 5A demonstrates NO removal in buffered solutions with and without 3 mM AA or 3 mM DHA. The time courses within the yellow circle are in hypoxic conditions (0.1 µM oxygen), while those outside the circle are equilibrated in air. In the presence of buffer alone, NO removal is faster in air (t_NO_  = 2.5 minutes, Table 1) due to reactions with oxygen in comparison to hypoxic conditions (t_NO_  = 16 minutes). AA has no effect in air, but actually slows hypoxic NO loss, whereas DHA speeds up NO removal under both conditions. This latter observation is not surprising as it is established that DHA and MDHA scavenge NO [46], [47]. The slowing of NO loss in hypoxia is probably due to reduction of nitrite (formed from NO oxidation during the experiment) by AA, which has been characterized in many systems [48], [49], [50] and is discussed in more detail below.

The experiments in Figure 5B were designed to test the effects of MDHAR/NADH and AAOx on NO scavenging. As described above, reactions with these enzymes were measured in air and under hypoxic conditions (in the yellow circle). These enzymes had few individual effects on NO scavenging. Under hypoxic conditions, the important variable was the presence or absence of AA. If AA is present under hypoxic conditions, NO removal is slightly slower. In air, AA has little effect, and only AAOx (in the presence of AA) had a clear effect on the time course for NO removal; it generated a scavenging time course resembling that for DHA (Figure 5A), which differs from the others by a slightly faster t_NO_, and also by the more-rapid removal of the final 20% of NO. We suspect that this is due to production of MDHA by AAOx as supported by the data in Figure 4B, showing that addition of NO removed the EPR signal associated with MDHA. There are also previous reports of direct scavenging of NO by MDHA [46], [51].

To test the effect of nsHb on NO scavenging in the conditions above, 5 µM ferric rice nsHb was included in reaction mixtures containing AA with and without MDHAR/NADH (Figure 5C). The lower concentration of nsHb compared to NO requires continued nsHb reduction to achieve complete NO scavenging. As a control for NO scavenging under these circumstances, time courses for scavenging by nsHb and ferredoxin reductase (Fdr)/NADH (a system characterized previously for rice nsHb [37], [52]) were also measured (dashed and dotted green lines). The incorporation of rice nsHb into these various AA-containing conditions had little effect on t_NO_ values (Table 1). As was the case for the reactions in Figure 5B, the only parameter that generally affected NO removal was the hypoxic presence of AA (which slowed NO removal slightly).

The more subtle effects of NO scavenging in air are clearer in the expanded comparison of the aerobic reactions (Figure 5D). Compared to AA alone, the addition of nsHb has no effect (thin black lines). Addition of AAOx (which produces MDHA and DHA) or DHA slightly decreases t_NO_ (red lines), and increase the speed of complete (100%) return to baseline compared to AA alone. MDHAR actually increases t_NO_ slightly, probably because it prevents formation of MDHA/DHA, which are better NO scavengers than AA [46], [47]. The presence of nsHb along with MDHAR has no effect on scavenging.

### Production of NO by AA and nitrite

**Figure 6:**
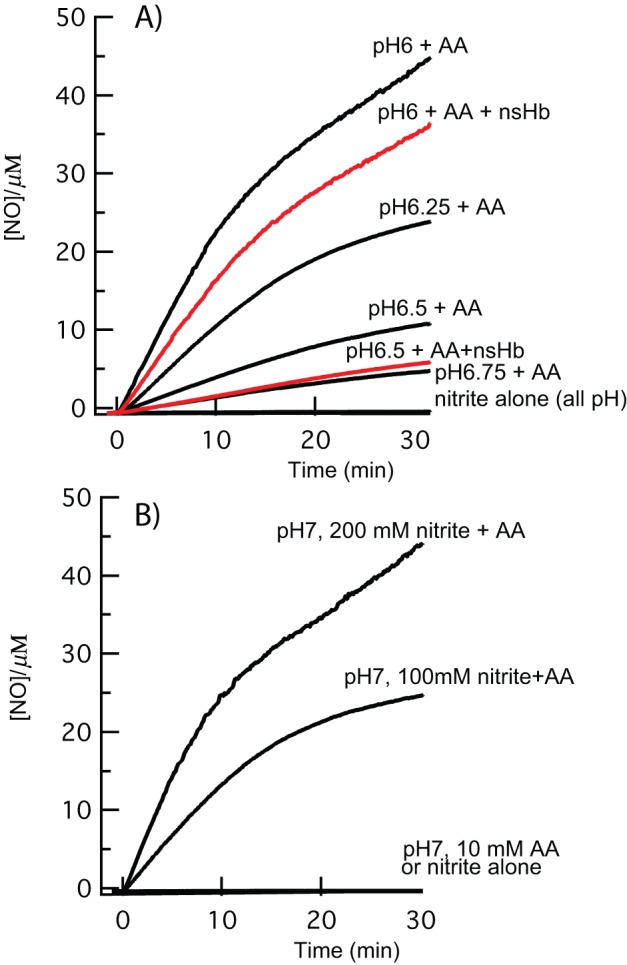
Production of NO from nitrite and AA. NO production from solutions of nitrite (10 mM unless otherwise indicated) were stimulated by addition of AA at pH values ranging from 6 to 7. A) NO production is measurable at pH 6.75 and increases with decreasing pH. When 5 µM nsHb is included (red traces) there is a decrease in the level NO proportional to the Hb concentration. B) The amount of NO produced is directly related to nitrite concentration, as is evident from the high levels produced even at pH 7 when nitrite concentration is very high.

The experiments above have tested the role of AA in mediating NO scavenging by nsHb. But AA could have other roles in NO metabolism. As nitrite concentrations rise, and pH drops, conditions inside hypoxic plant cells favor NO production by the direct reduction of nitrous acid by AA [21], [48]. Our final experiment investigating the role of AA in NO metabolism was designed to test whether such conditions could yield measurable NO concentrations *in vitro*. Figure 6 demonstrates results from reactions in which AA was added to solutions of nitrite buffered at pH values ranging from 6 to 7, under hypoxic conditions. In each experiment (unless otherwise labeled), 10 mM nitrite solutions were equilibrated with the NO electrode at the desired pH, and then AA (to make the reaction 3 mM AA) was added (or not, as indicated) at time 0. Nitrite alone produces no NO emission at these pH values (Figure 6A). However, as pH is lowered to 6.75, measurable (∼5 µM) NO is produced from the 10 mM nitrite samples over a thirty-minute period. As pH is lowered to 6.5, 6.25, and 6, NO emission increases accordingly to values approaching 50 µM over this time period. NO is not detected at pH 7 from 10 mM nitrite, but when nitrite is increased to a very high (100 and 200 mM) concentrations, NO is released rapidly, reaching concentrations near 50 µM after 20 minutes (Figure 6B).

To test the role of nsHb under these conditions, 5 µM rice nsHb was included in the incubation mixture of separate reactions at pH 6 and pH 6.5. The amount of NO produced in these reactions (compared to the respective ones lacking nsHb) was decreased by about 5 µM, as would be expected by stoichiometric NO binding to the nsHb. As nsHb concentration in cells is thought to be low (<1 µM), direct binding of NO would have little effect on NO concentrations.

## Discussion

NO metabolism in plants has been linked to functions in signaling, toxicity during hypoxia, and even minor manipulations of the mechanisms of production and scavenging are believed to have potentially important affects on downstream biochemistry [20], [53]. The results presented here are an important test of the hypothesis that the ascorbate cycle mediates nsHb scavenging of NO during hypoxia, and provides a plausible explanation for NO production during hypoxia based only on increased nitrite concentrations, lowered pH, and the presence of AA. The discussion below integrates our results into three subjects of current interest in plant physiology. The first addresses interactions between AA and nsHbs in the context of NO scavenging; the second concerns interactions between AA and nitrite in hypoxic tissues, and the third the physiological role of nsHbs.

### The role of AA and MDHAR in nsHb-mediated NO scavenging

The AA/MDHAR hypothesis for NO scavenging states that nsHb uses the NOD reaction to scavenge NO (Figure 1B) [39]. The mechanism for the oxidative half of the reaction is not in question, as ferrous nsHbs readily bind oxygen [54], and oxy-nsHb rapidly reacts with NO to form nitrate [30]. The work presented here addresses the unique hypothesis for the reductive half of the NOD reaction, which suggests that AA directly reduces nsHb, and that MHDAR facilitates this activity by reducing the product, MDHA, back to AA using the reduction power of NAD(P)H. Our results suggest that AA could contribute to slow nsHb reduction, but they do not lend support to a contributing role for MDHAR. Nor do they suggest that AA stimulates NO scavenging by nsHbs under hypoxic or normoxic conditions.

Most of our experiments were carried out starting with a reduced ascorbate pool (in the form of AA rather than MDHA or DHA), which mimics hypoxic cells [11]. It is possible that MDHA would have a larger effect on nsHb reduction if the ascorbate pool were oxidized (such as upon re-aeration of hypoxic plant tissue), because it could shift the pool back toward AA, which has a higher rate of reduction of nsHb than DHA (Figure 2). However, without an efficient reductase for nsHb, the direct reaction of NO with DHA would serve as a better NO scavenger than the nsHb/AA system. Thus, our results do not support a role for NO scavenging by nsHb using a NOD-based mechanism.

### Interactions between nitrite and AA: a potential source of NO in hypoxic plant cells

The sources of NO in plant cells are not entirely clear, and there are several hypothesized mechanisms of NO production [23], [53]: 1) reduction of nitrite by nitrate reductase; 2) a plasma-membrane bound nitrite:NO reductase; 3) hypoxic mitochondria; 4) anaerobic xanthine oxidase; and 5) nsHb reduction of nitrite [55], [56], [57]. Only for reactions 1) and 5) have purified plant proteins been shown to produce NO in the laboratory.

Nitrate reductase produces NO by acting on nitrite rather than nitrate [58], [59]. This occurs when nitrite concentrations rise, such as when nitrite reductase is inhibited at lowered pH during hypoxia [58], [60], [61]. Ferrous nsHb produces NO by reducing nitrite under anaerobic conditions. *In vitro*, the resulting NO binds tightly to the remaining ferrous nsHb and rapidly inhibits further nitrite reduction [55], but low levels of NO release have been measured from this system [56].

Production of NO in plants from the direct reaction of AA and nitrite has been considered [59], but was discounted because of the low levels of nitrous acid (with a pKa of 3.4) that would be present at neutral pH. Our results affirm that NO production from AA and nitrite is directly proportional to nitrous acid concentrations. Thus, at pH values of 7, 6.75, 6.5, 6.25, and 6.0, and a starting nitrite concentration of 10 mM, one would expect nitrous acid concentrations of 2, 4, 8, 14, and 25 µM, respectively; these values are close to those observed for each experiment (Figure 6). Therefore, assuming AA is not limiting, one can estimate NO production from the nitrite concentration and pH. For example, at pH 6.5 and a nitrite concentration of 5 mM (as might be found in hypoxic plant roots), one would expect the production of 4 µM NO. Thus, even though the pKa for nitrous acid is relatively low, the nitrite concentrations present in hypoxic roots are sufficient for the production of significant levels of NO, especially as the pH drops below 7.

### How do these results impact our hypothesis for nsHb function?

The preponderance of data supports a function for nsHbs related to NO metabolism and hypoxia. The two clearest possibilities are NO scavenging using the NOD mechanism, and NO production from nitrite. The direct chemical evidence in support of NOD function for nsHbs is not strong. These properties are not unique to nsHbs, are in fact common to most hemoglobins [37], and thus do not provide a stringent test for the potential of NOD as a specific physiological function.

Direct chemical evidence in support of NO production is stronger, as nsHbs are much better nitrite reductases than other hemoglobins [55], [56]. However, this story is incomplete, as it is not clear what happens to NO after it is produced. Some options are 1) it simply binds the remaining ferrous nsHb and prevents further reaction, 2) NO is somehow released, contributing to the NO pool in the hypoxic cell, or 3) the NO is further reduced in a dissimilatory manner in support of hypoxic respiration. Option 3) would result in net NO scavenging, as it proposes that whatever NO is produced by the nsHb is used in subsequent reactions.

A recent investigation of nsHbs demonstrated NO production and release by *Arabidopsis* leaves in wild type, nsHb knock-down, and nsHb over-expressing plant lines [24]. Only very low levels of NO was observed in an environment above 1% oxygen for any of the lines, but as oxygen levels were lowered below 1%, the knock-down line lost NO at elevated levels compared to the wild type and over-expressing lines. The most NO was released under total anoxia (0% oxygen) in the knock-down line, but all lines released significant amounts of NO under that condition. These data are important because they reveal NO production and release on a large scale during hypoxia even for wild-type plants, and show that nsHbs are involved. They were interpreted in support of NOD function by nsHbs because the nsHb knock-down line released the most NO during hypoxia and anoxia.

If nsHb were simply using the NOD mechanism to scavenge NO, the anoxic conditions should have shown no difference between plant lines, as none would be capable of NO scavenging in the absence of oxygen. Alternatively, these data could be interpreted as nsHb removal of NO using a dissimilatory reductive mechanism in which electrons are shuttled to nitrite, NO, and potentially hydroxylamine, to make ammonia (this is one possibility for Option 3 above). Dissimilatory reduction of NO could benefit the plant by consuming more respiratory and/or glycolytic electrons than would NO oxidation using NOD, and it would not require the consumption of oxygen, which could be put to better use in mitochondrial respiration. In support of this hypothesis is the fact that ferrous nsHb is a very effective hydroxylamine reductase, which forms ammonia under anaerobic conditions [62]. However, for nsHbs to serve as dissimilatory reductases would require mechanisms for producing hydroxylamine and over-coming NO-inhibition of ferrous nsHb, as well as a mechanism for reduction of ferric nsHb. Thus, further biochemical experiments with nsHbs and inorganic nitrogen species, and assays for hypoxic nitrogen utilization in plants lacking nsHbs will be critical for testing this hypothesis.
